# Protective Role of Nutritional Plants Containing Flavonoids in Hair Follicle Disruption: A Review

**DOI:** 10.3390/ijms21020523

**Published:** 2020-01-14

**Authors:** Eleonora Bassino, Franco Gasparri, Luca Munaron

**Affiliations:** 1 Department of Life Sciences and Systems Biology, University of Turin, Via Accademia Albertina 13, 10123 Torino, Italy; luca.munaron@unito.it; 2 Department of Pharmacy, University of Salerno, 84010 Salerno, Italy; info@gasparrifranco.it

**Keywords:** nutraceuticals, alopecia, *flavonoids*, Panax *ginseng* C.A. Mey., *Malus pumila* Mill cultivar Annurca, Caffeine, *Allium sativum* L., Camellia sinensis (L.) Kuntze, *Rosmarinum officinalis* L., *Capsicum annum* L.

## Abstract

Hair loss is a disorder in which the hair falls out from skin areas such as the scalp and the body. Several studies suggest the use of herbal medicine to treat related disorders, including alopecia. Dermal microcirculation is essential for hair maintenance, and an insufficient blood supply can lead to hair follicles (HF) diseases. This work aims to provide an insight into the ethnohistorical records of some nutritional compounds containing flavonoids for their potential beneficial features in repairing or recovering from hair follicle disruption. We started from a query for “alopecia” OR “hair loss” AND “*Panax* *ginseng* C.A. Mey.“ (or other six botanicals) terms included in Pubmed and Web of Sciences articles. The activities of seven common botanicals introduced with diet (*Panax* *ginseng* C.A. Mey.*, Malus pumila* Mill cultivar Annurca*,* Coffea arabica, *Allium sativum* L*.,* Camellia sinensis (L.) Kuntze*, Rosmarinum officinalis* L., *Capsicum annum* L.) are discussed, which are believed to reduce the rate of hair loss or stimulate new hair growth. In this review, we pay our attention on the molecular mechanisms underlying the bioactivity of the aforementioned nutritional compounds *in vivo*, ex vivo and in vitro studies. There is a need for systematic evaluation of the most commonly used plants to confirm their anti-hair loss power, identify possible mechanisms of action, and recommend their best adoption.

## 1. Introduction

Androgenic alopecia (AGA) is a common form of hair loss (HL), affecting men and women at different ages [1,2]. In AGA, androgens are involved in the reduction of anagen phase, inducing an increment of the number of hair follicles (HF) in the catagen and telogen phases as well as delaying the telogen-to-anagen transition [3].

Generally, androgens affect several functions of the human skin, including sebaceous gland proliferation and differentiation, hair growth (HG), and wound healing [4]. Though other hormones, such as thyroid hormones and glucocorticoids, are involved in the process, androgens act as the master players in human HG.

AGA is associated with high levels of dihydrotestosterone (DHT) released from testosterone by the enzymatic activity of 5-alpha reductase (5α-R). DHT binds to the androgenic receptor (AR) to form a hormone–receptor complex. DHT drastically reduces anagen phase, enhancing apoptosis of hair cells, miniaturizing the HF, and causing HL [5]. The expression of both 5α-R and AR is enhanced in patients with AGA [6].

Androgens activity is also explained by in vitro experiments, where androgen stimulation of scalp follicle dermal papilla cells (FDPC) triggers TGF-1 production [7]. The synthetic androgen R1881 suppresses keratinocyte growth in the co-culture of AR-overexpressing human FDPC obtained from AGA and normal human keratinocytes through androgen-inducible TGF-1 [8,9]. As TGF-1 is known to be a catagen inducer in hair cycling, it likely plays a role in the early catagen induction seen in AGA [10]. Furthermore, TGF-2, the Wnt antagonist dickkopf-1, and IL-6 have been identified as androgen-inducible negative regulators for AGA in in vitro experimental models [11,12].

Dermal microcirculation (DM) is essential for hair maintenance by delivering growth factors, nutrients, cytokines, and other bioactive molecules, as well as by removing waste metabolic products. DM is composed of a branching network of microvessels (<100 µm diameter) classified as arterioles, venules, and capillaries [13]. DM has a remarkable capacity for adaptation to its cellular environment and continuous interaction with the systemic circulation [13]. DM is essential for the maintaining of skin homeostasis and includes different types of vessels, such as arterioles (from 17 to 26 μm in diameter), meta-arterioles (10 to 20 µm), capillaries (8 to 10 µm), and venules [14].

The circulatory support during anagen phase suggests its association with the high metabolic activity of HF matrix cells, and insufficient blood supply can lead to HF diseases [15]. Accordingly, proangiogenic vascular endothelial growth factor (VEGF) regulates HG and follicle size [15].

**Figure 1:**
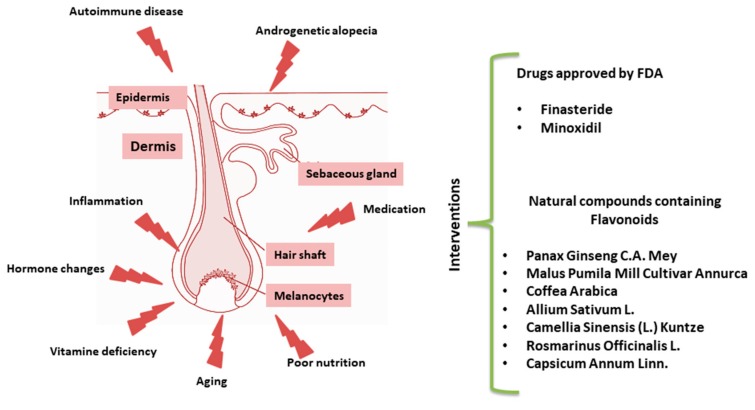
Schematic representation of hair follicle (HF) disruption. List of pharmacological drugs approved by Food and Drug Administration (FDA) or natural interventions.

Based on this mechanistic knowledge, several drugs have been formulated for the treatment of alopecia over the last decade [1]. Here, we provide an overview of the currently available literature devoted to alternative natural dietary treatments for HL (Figure 1). Among others, a recent work paid attention to Mediterranean diet supporting the hypothesis that some fresh herbs and salad, rich in phytochemicals such as carotenoids and polyphenols, may reduce the risk of AGA [16].

In the present review, we started for a query for “alopecia” OR “hair loss” AND “*Rosmarinum officinalis* L.” (and other 6 botanicals) in ARTICLE (Title/Abstract/Keyword) in PUBMED and Web of Sciences database.

## 2. Results

### 2.1. Traditional Medical Treatments

**Figure 2:**
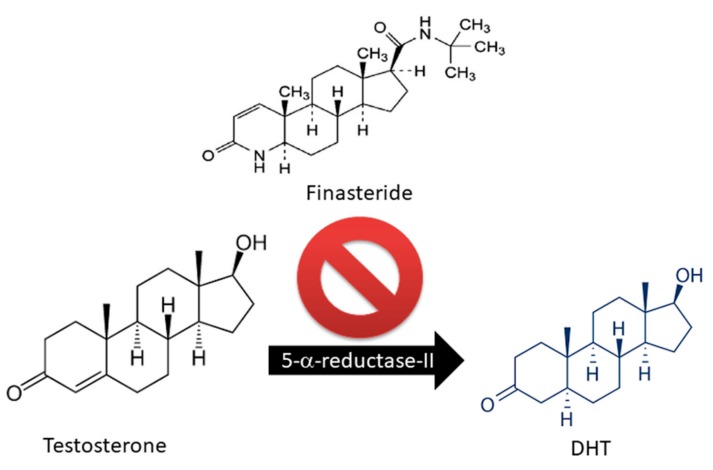
Finasteride action on Testosterone metabolism: block of 5-alpha reductase activity.

Only two drugs are currently approved by the U.S. Food and Drug Administration (FDA) for the treatment of AGA: Finasteride and Minoxidil (MXD) [17,18,19]. Finasteride is largely used as an oral drug for the treatment of androgen-related hair disorders due to its inhibitory activity on 5α-R [18,19,20] (Figure 2).

**Figure 3:**
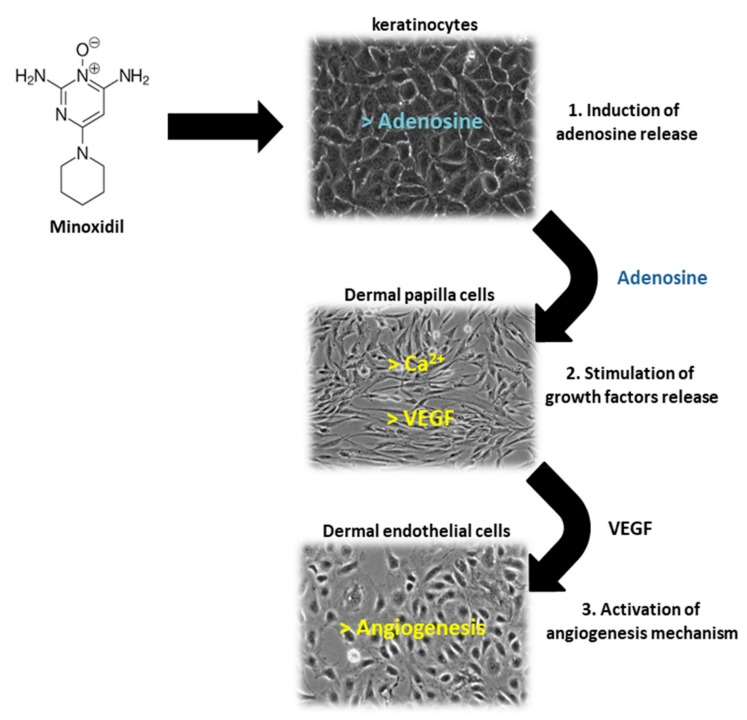
Schematic representation of Minoxidil (MXD) action on different cells of HF: keratinocytes, dermal papilla cells and dermal microvascular endothelial cells. (1) MXD induces the release of adenosine by follicle keratinocytes, (2) adenosine stimulates the release of growth factors (VEGF) by follicle dermal papilla cells (FDPC), (3) VEGF promotes angiogenesis mechanism in dermal microvascular endothelial cells.

Of the two 5α-R human isoenzymes [19,20], type I predominates in the liver and skin including the scalp [21,22], whereas type II is present in HF [23], as well as the prostate and genitourinary tract [22]. Finasteride was approved in 1997 at a dosage of 1 mg/day for use in adult men with mild-to-moderate AGA. In addition to finasteride, a 5α-R2 inhibitor, a novel molecule called dutasteride has recently been developed which is able to affect both 5α-R1 and 5α-R2. Oral dutasteride (0.5 mg/day) is another option, but there are no studies that compare its efficacy to that of finasteride [24]. MXD was originally developed as an antihypertensive agent, but attracted interest as a potential HL therapy; indeed, patients treated with MXD developed generalized hypertrichosis [25]. MXD is a potassium channel opener [17] and is used as topical application for male pattern baldness [9]. The mechanism of action of MXD is not completely defined, but HG promotion is suggested to occur with an increased production of several growth factors (i.e., IGF and VEGF), improving DM [17,25,26] (Figure 3). In vitro studies of HG evidenced that hair cultures grown in the presence of MXD increase their viability, whereas controls undergo necrosis.

### 2.2. Natural Alternative Interventions

Pharmacological approaches have their own side effects. For this reason, the medical attention on HF disruption has been recently focused on the discovery of new and safer remedies, often provided by natural therapies (e.g., *Serenoa repens* (*SR*) [27]. *SR* is an extract from the berries of the saw palmetto palm tree (American dwarf tree) containing phytosterols (β-sitosterol), fatty acids, β-carotene, and polysaccharides. *SR* acts as a competitive, nonselective inhibitor of both forms of 5α-R enzyme. The extract obtained from the berries of *SR* is rich in fatty acids (85–90%), and the other constituents include sterols rich in carotenoids, lipases, tannin, sugars, and beta-sitosterol. In particular, sitosterol and fatty acids are responsible for the inhibition of 5α-R [28]. Recently, we reported that *SR* affects dermal endothelial cells’ permeability and tight junction proteins content in the paracrine communication with follicle dermal papilla cells, providing a mechanistic background for its potential use to promote HF vascularization [29].

The increasing use of natural dietary products is based on their ability to prompt hair growth (HG) activity, to promote anagen phase, and to improve DM [30]. About this, recently, our laboratory tested the effects of three flavonoids (visnadin (VSD), hesperidin (HSP), and baicalin (BC)), on primary human dermal microvascular endothelial cells (HMVEC), comparing their effects with MXD. BC promoted endothelial proliferation, while VSD and MXD enhanced angiogenic potential. Moreover, only HSP increased VEGFR-2 phosphorylation [31].

Phenolic compounds and vitamins may give more specific support to the maintenance of HF health [32]. Vitamin C improves blood vessel formation, increasing blood flow in the scalp by stimulating VEGF synthesis [33]. Moreover, vitamin C potentiates the efficacy of therapeutic angiogenesis by cell transplantation [34]. Vitamin D receptors are intracellular receptors expressed in HF cells, essential for normal hair cycle and differentiation of the interfollicular epidermis. Homozygous knockout of murine vitamin D receptor results in the development of alopecia and near-total HL at 8 months [35]. The lack of vitamin D receptor is associated with reduced epidermal differentiation and HF growth. Deficiency of vitamin B7 (biotin) induces alopecia and other skin alterations such as dermatitis and conjunctivitis. Accordingly, biotin supplementation is effective for HL in clinical trials [36]. In addition, a large variety of amino acids has been considered for the treatment of HL. In particular, cystine and lysine effects have been widely characterized in humans. Oral administration of L-cystine (70 mg) used in combination with retinol increases both hair density and anagen rate [37]. The combination of L-cystine, histidine, and copper significantly increases the total hair count after 50 weeks in patients with AGA [38]. In summary, L-cysteine plays a central role in the maintaining of hair health. Another important amino acid characterized for its HG promotion properties is L-lysine, an essential amino acid found in meat and eggs. In patients with chronic telogen affluvium, supplementation with L-lysine, iron, vitamin B12, vitamin C, biotin, and selenium (Florisene^®^; Lambers Healthcare Ltd., Kent, UK) resulted in a significant reduction of hair shedding after 6 months [39].

Polyphenolic phytochemicals also display several pharmacological properties and serve as a rich reservoir for drug discovery. Currently, a limited number of studies reported the role of flavonoids in patients with alopecia [40].

Silibinin, a secondary metabolite from *Silybum marianum*, is an effective antioxidant that prevents various cutaneous problems. Recently, silibinin treatment was reported to enhance spheroid formation of FDPC through the activation of AKT and Wnt/β-catenin-signaling pathways [41]. Another important flavonoid widely used as traditional Chinese herbal medicine is BC. BC is commonly used for HL treatment, but the precise molecular mechanism remains unknown. In mouse FDPC, BC stimulates the expression of Wnt3a, Wnt5a, frizzled 7, and disheveled 2, whilst inhibiting the Axin/casein kinase 1α/adenomatous polyposis coli/glycogen synthase kinase 3β degradation complex and inducing the Wnt/β-catenin-signaling activation. Moreover, BC increases the alkaline phosphatase levels in FDPC, a process associated with the activation of Wnt pathway [42]. BC is mitogenic for human FDPC, where it prevents nuclear translocation of the AR driven by DHT. These results suggest that BC could be used as an active ingredient for treating androgen-related disorders, such as AGA [43].

Improvement of AGA was also observed after the topical application of *Sophora flavescens* Aiton (Leguminosae) extract [44]. *Sophora flavescens* Aiton is a plant of traditional Chinese medicine. A number of experimental observations have shown that *Sophora flavescens* root extract has vasodilatory, antibacterial, antiandrogen, and antiulcer effects, making it a promising tool for an effective HL treatment [44]. *Sophora flavescens* extract induces IGF-1 and FGF-7/KGF expression in cultured FDPC and strongly inhibits 5α-R [45].

Prevention of alopecia areata (AA) was also observed after the treatment with quercetin. It may be effective in both prevention and treatment for AA in the C3H/HeJ model, even if further clinical studies are required [46].

In vivo studies evidenced that dietary supplementation with soy oil and soy-derived phytoestrogen genistein significantly increases resistance to AA. Dietary soy oil content and soy-derived phytoestrogen genistein increase resistance to AA onset in C3H/HeJ mice. The principal isoflavones in soy beans are genistein (4′,5,7-trihydroxyisoflavone) and daidzein (4′,7-dihydroxyisoflavone). Soy oil content can alter susceptibility to AA induced by skin grafting, with a potential key role for and soy-derived genistein [47].

This work aims to provide an insight into the ethnohistorical records of some nutritional compounds containing flavonoids for their potential beneficial features in repairing or recovering from hair follicle disruption.

### 2.3. Botanicals Containing Flavonoids

Here, we consider seven natural compounds (*Panax ginseng* C.A. Mey.*, Malus pumila* Mill. cultivar Annurca*,* Coffea arabica L.*, Allium sativum* L*.,* Camellia sinensis (L.) Kuntze*, Rosmarinum officinalis* L., and *Capsicum annum* L.), containing flavonoids that are commonly introduced with diet and whose in vivo, ex vivo, and in vitro biological activities are reported by recent specialized literature.

#### 2.3.1. *Panax ginseng* C.A. Mey.

*Panax ginseng* C.A. Mey. is a traditional Chinese remedy well-known worldwide and known by the popular name *ginseng*. The three key ingredients of ginseng are saponins, polysaccharides, and phenolic compounds [48]. Saponins are recognized as primary pharmacologically active components [49]. Ginsenoside (is the alternative name of saponins) are categorized into two different groups, based on their chemical structure, known as oleanane type (five ring structure) and dammarane type (four ring structure). Ginseng contains two types of polysaccharides: one of them is composed of starch like glucan known as neutral polysaccharide, and the other is an acidic polysaccharide [50]. Acidic polysaccharide has enhanced antitumor and antioxidant properties as compared with neutral polysaccharides [50]. Ginseng also contains non-water-soluble polyacetylenes such as the panoxynol-polyacetylene [50]. Polyacetylenes do show effects against cancerous cells, but in vivo there is no proven fact presently owing to their chemical nature [51]. Recently, research has demonstrated that ginseng acts as a chemo-preventive agent owing to its anticancer properties. In general, red ginseng oil (RGO) exerts anti-inflammatory [50], antioxidant, and hepato-protective activities [50]. The wild-growing or cultivated ginseng root, which is collected in the fall, is officially listed in the Chinese Pharmacopoeia and is used as a tonic. *Panax ginseng* C.A. Mey. has been revealed to promote HG [52,53,54]. Treatment of mice with RGO upregulates Wnt/β-catenin and Shh/Gli-pathways-mediated expression of genes such as β-*catenin, Lef-1, sonic hedgehog, Cyclin D1, and Cyclin E* [55]. In addition, RGO and its major components downregulate TGF-β [56,57,58] and enhance antiapoptotic protein Bcl-2 expression [55]. Matsuda H. et al., (2003) reported the promotion of HG by ginseng radix on cultured mouse vibrissal HF [59]. Korean red ginseng (KRG), in combination with topical MXD, is more effective than MXD for the promotion of HG in human clinical studies [60]. Ginsenoside is able to increase the hair shaft length and hair existent time, which is similar to the action of MXD in in vivo experimental models (topical treatment in nude mice), moreover, it stimulates hair shaft elongation in ex vivo cultures of vibrissa HF and suppresses TGF-β-induced phosphorylation of ERK in HeLa cells [61]. It also promotes the proliferation of human dermal follicle papilla cells and keratinocytes and enhances hair anagen induction or acceleration of HG in mouse [61] (See Table 1). In chemotherapy-induced alopecia, KRG protects against premature catagen development through modulation of p53 and Bax/Bcl2 expression. Finally, ginseng exerts mitogenic and antiapoptotic effects in outer root sheath keratinocytes [62,63].

#### 2.3.2. *Malus pumila* Mill. cultivar Annurca

*Malus pumila* Mill. cultivar Annurca (*Annurca apple*), a native of Southern Italy, displays higher content of oligomeric procyanidins, such as procyanidin B2, compared with more common apple samples including Red Delicious, Granny Smith, Pink Lady, Fuji, and Golden Delicious [64,65]. In general, apples are the most deeply studied and consumed fruits in northern Europe and North America. Phytochemical studies have shown that apple contains various components including polysaccharides, tri-terpenoids, phytosterols, phenols, and other components such as protein [66], vitamins (A, C, and E), α-carotene, and metal elements (i.e., iron, magnesium, calcium, zinc, manganese, sulfur, copper, and potassium) essential for human health [66]. Apples also contain different polyphenols in different tissues. In particular, variety and contents of polyphenols in apple peel are relatively high, although there are different polyphenols in apple: phenolic acid and flavonoids are reported to be the most important constituents [66] (see Table 2).

Clinical trials provide strong evidence supporting the ability of procyanidin B2 contained in Annurca apple extract to improve HG and skin quality, as well as to increase hair density, weight, and keratin content [67]. Procyanidin B2 acts on keratin biosynthesis in a nontumorigenic human keratinocyte cell line, suggesting its nutraceutical power on human HG and tropism [66,67]. Polyphenols of Annurca apple regulate murine HF metabolism, inhibiting several NADPH-dependent reactions and stimulating mitochondrial respiration, β-oxidation, and keratin production [66,67] (see Table 2). The same components protect murine HF from taxane-induced alopecia, a common side effect of conventional chemotherapy [68].

#### 2.3.3. Allium sativum L.

*Allium sativum* L. (garlic) originates from central Asia (over 6000 years ago) and has long been a staple food in the Mediterranean region, as well as a frequent seasoning in Europe and Africa [69]. Moreover, it represents a part of the daily diet for most of the human population. Fresh garlic contains water, fiber, lipids, proteins, carbohydrates (such as fructose), vitamins (mainly C and A), minerals (such as potassium, phosphorus, magnesium, sodium, iron, and calcium), phytosterols, and phenolic derivatives, as well as organic sulfur compounds. On the basis of solubility, its chemical composition can be divided into two large groups: 1) compounds of lipid-soluble allyl sulfur (such as diallyl sulfur (DAS), diallyl disulfide (DADS), and diallyl trisulfide (DATS)) and 2) water-soluble compounds, such as of g-glutamyl S-allylcysteine (SAC) and S-allyllmercaptocysteine (SAMC) [70,71]. *Allium sativum* L. has been widely used throughout history for its prophylactic and therapeutic effects. It is still used in folk medicine all over the world to treat several diseases [72]. *Allium sativum* L. is widely known for its biological properties and plays an important role as an antioxidant [73]. *Allium sativum* L. modulates lipid metabolism, and its consumption is associated with lowering blood cholesterol levels. *Allium sativum* L. exerts a significant effect on the cardiovascular system where it reduces blood pressure, inhibits platelet aggregation, and exerts antioxidant and fibrinolytic activities [73]. Moreover, *Allium sativum* L. significantly decreases HMG-CoA reductase activity and may have an effect on the level of cholesterol hydroxylase and other enzymes. Its oral administration is effective on DM [74]; in particular, a randomized placebo-controlled, double-blinded study demonstrated that administration of *Allium sativum* L. powder for 5 h significantly improves capillary skin perfusion in 55% of healthy volunteers [74]. *Allium sativum* L. is also involved in protection from photodamage and cellular senescence in UVB-exposed human keratinocytes [75] (Table 3). *Allium sativum L.* was recently considered among herbal medicines for clinical research in the field of AA treatment [76]. In a double-blinded, randomized controlled trial of subjects affected by AA, garlic gel significantly increased the therapeutic efficacy of topical betamethasone [77].

#### 2.3.4. Coffea arabica L.

Caffeine from Coffea arabica L. is a xanthine (purine) alkaloid also found in *Paullinia cupana* Kunth (Guarana), *Ilex paraguariensis* A.St.-Hil. (yerba maté), *Cacao minar* Gaertn. (Cacao), and several species used to make tea [78]. The type of caffeine is strictly related to the social habits and the culture of regions [78]. In green coffee, the largest lipid fraction occurs in the oil of the bean endosperm. There is also a small amount of wax in the outer layers of the bean. Coffee oil consists of triacylglycerols, phospholipids, sterols, tocopherols, and diterpenes [79]. Caffeine effects are mainly mediated by the inhibition of phosphodiesterase, which causes an increased intracellular adenylate cyclase activity, enhancing the cyclic 3′,5′-adenosine monophosphate (cAMP) levels, therefore providing higher energy levels to promote increased metabolic activity and cell proliferation. Coffee has been studied for a long time in relation to its cultural and social impact, and some of its components have been isolated. Products containing caffeine are promising tools for potential treatments for AGA both in vitro and in vivo models. In ex-vivo models, they stimulate human HG of HF obtained from patients affected by AGA [80]. Specifically, a work by Fisher et al. (2007) showed caffeine enhancement of hair shaft elongation, prolonging anagen duration and hair matrix keratinocyte proliferation; moreover, female HFs show a higher sensitivity to caffeine than male HFs [80]. Caffeine counteracts testosterone-enhanced TGF-2 protein expression in male HFs and reduces TGF-2 expression in female HFs, while it enhances intrafollicular IGF-1 protein expression in both sexes [80]. Accordingly, it enhances hair shaft elongation, prolongs anagen duration, and promotes the proliferation of hair matrix keratinocytes [81] (Table 4). In men affected by AGA, topical application of caffeine may be considered more effective than MXD 5% topical solution [82]. In female subjects, shampoo containing caffeine significantly prevents HL [82].

#### 2.3.5. Camellia sinensis (L.) Kuntze

Camellia sinensis (L.) Kuntze, is an evergreen, perennial plant, including cross-pollinated tree species native to China and Southeast Asia, very important for human well-being [83]. Tea, a product of the plant Camellia sinensis (L.) Kuntze, is one of the most consumed beverages in the world. Green, black, and oolong teas are made from the same plant but are processed differently, depending on their degree of fermentation [84]. Several human observational and intervention studies have found beneficial effects of tea consumption on neurodegenerative impairment, such as cognitive dysfunction and memory loss [83]. Actually, some reports have demonstrated that catechins are the major components in leaves that acted as agonists of the nuclear receptor protein peroxisome proliferator-activated receptor gamma (PPAR-c); therefore, they could be considered as current pharmacological targets for the treatment of type 2 diabetes mellitus (T2DM) [85]. The rich saponins and catechins in *Camellia sinensis* (L.) Kuntze seeds have been known to lower body weight and serum lipid levels [86,87]. Camellia sinensis (L.) Kuntze leaves contain catechins, such as epigallocatechin-3-gallate, as well as quercetin, the aflavins, the arubigins, the anine, caffeine, chlorogenic acid, and gallic acid. Its beneficial anticancer and antioxidant effects are mediated by epigallocatechin-3-gallate (EGCG), the major constituent of polyphenols [87,88]. p38 and JNK-MAPK pathways are essential for EGCG induction of p57 and caspase 14 in keratinocytes, reducing psoriasiform lesions in the flaky skin mouse model [89] (see Table 5). Recently, EGCG was proposed for the prevention or treatment of AGA by selectively inhibiting 5**α**-R activity [90]. It reveals as a potent inhibitor in cell-free environment but not in whole-cell assays of 5α-R. Replacement of the gallate ester in EGCG with long-chain fatty acids produced potent 5α-R inhibitors that were active in both cell-free and whole-cell assay systems [90]. EGCG stimulates human HG via its proliferative and antiapoptotic effects on FDPC and may prolong anagen stage [91]

#### 2.3.6. Rosmarinus officinalis L.

*Rosmarinus officinalis* L. (RO) is a common evergreen, aromatic shrub that grows in several areas of the world. RO is known for its nutritional values and pharmacological properties that made it renowned in traditional medicine [92,93]. RO extract significantly prevents food deterioration when added to products such as dairy products, meat, fish, oil dressing, and frying oil; moreover, it shows better efficiency than conventional preservatives [94]. The main active compounds responsible for these functions in RO are carnosic acid (CaA), carnosol, and rosmarinic acid [95]. Although CaA has the most potent antioxidant and antimicrobial activity among these active compounds [94,95,96], its deployment in water-based food systems is limited by its low solubility [97]. RO is used in cosmetic products and displays different properties, including enhancement of microcapillary perfusion [97]. Indeed, in the study by Panahi et al. (2015), patients with AGA were randomly assigned to rosemary oil or MXD 2% for 6 months, and the results obtained evidenced no significant change in the mean hair count at the 3 month endpoint, neither in the RO nor in the MXD group. Conversely, the authors reported that both groups reported a significant increase in hair count at the 6 month endpoint [97].

In the work of Borrás-Linares (2014), it was reported that an improvement of blood circulation and vascularity induced by RO helps the regeneration of HF similarly to MXD [98]. A randomized comparative trial provided evidence for RO efficacy in the treatment of AGA [97]. Topical administration of RO leaf extract (2 mg/day/mouse) improved HG when topically administrated to C57BL/6NCrSlc mice [99] and promoted prostate cancer cell line LNCaP proliferation in in vitro models. Moreover, RO leaf extract showed inhibitory activity on testosterone 5α-R [99] (Table 6).

#### 2.3.7. Capsicum annum L.

Capsicum annuum L. originated in the area bordered by the mountains of Southern Brazil, Bolivia, Northern Argentina, and Paraguay [100]. It is one of the major vegetable and spice crops cultivated worldwide for its nutritional value and spicy nature.

In addition to their sensory features, such as pungency, aroma, and color, peppers are important sources of bioactive compounds, such as vitamins (C and E, provitamin A) and phenols compounds. The content of these phytochemicals changes with the metabolic and chemical processes; therefore, sampling and storage conditions (i.e., T °C <7.5 °C, oxygen depletion, absence of light, or ∼70% relative humidity) may be controlled in order to produce a high-quality plant material for its characterization and further use [101]. The amount of bioactive compounds is associated with the fruit part (placenta, pericarp, and seeds), the cultivar or variety, the ripening stage, the climatic and storage conditions, as well as the processing practices [102]. Carotenoids are usually the major phytochemicals found in pepper varieties, which add high commercial value to these fruits in terms of color and antioxidant properties and flavor characteristics, among other bioactivities. Capsaicinoids are mainly found in hot peppers and are responsible for the pungency of these varieties. Peppers are also rich in phenolic compounds, such as flavonoids and phenolic acid derivatives, as well as vitamins (A and C) and minerals (i.e., iron, calcium, and manganese), highly contributing human nutrition [103].

Experimental observations strongly suggest that the combination of capsaicin and isoflavone, two bioactive compounds included in the crop, significantly increase IGF-I production in HF, promoting HG [103,104]. In particular, capsaicin binds to transient receptor potential cation channel subfamily V member 1 (TRPV1), also known as the vanilloid receptor 1, increasing the release of calcitonin peptide (CGRP) from sensory neurons and finally raising IGF-I production [105]. Intradermal injection of capsaicin induces anagen phase in mice [105]. A new antibaldness agent based on Interconnected PolymerS TeChnology is an effective strategy for the delivery of bioactive molecules such as Origanum vulgare leaf extract, Camellia sinensis leaf extract, and Capsicum annuum fruit extract, and their combined activities are mediated by sensory neuron activation in the skin [106] (Table 7).

When capsaicin was injected intradermally in C57BL/6 mice with all follicles in the telogen phase of HF cycle, it induced significant hair growth (anagen) in the back skin of telogen mice, which was associated with substantial mast cell degranulation [107].

## 3. Discussion

HG is a cyclically controlled process formed by three distinct phases in mammals, respectively called anagen (growing phase), catagen (regressing phase), and telogen (resting phase). During catagen, HF regression is a tightly coordinated process characterized by apoptosis and terminal differentiation of the proximal epithelial HF, perifollicular proteolysis, and matrix remodeling [108,109]. The development of HG is influenced by a variety of growth factors and cytokines, as well as the keratinocyte growth factors interleukin-1 and TGF-β [107].

HL is a symptom of a variety of several forms of alopecia, including AGA, AA, and telogen effluvium [110]. Recognized treatment involves the use of drugs that are effective only upon continued administration and are not free from side effects [110,111,112,113]. Some patients may find MXD use unhandy or expensive. Moreover, potential cardiovascular side effects may discourage its prescription for the treatment of HL by dermatologists [114,115]. In relation to finasteride, controversy surrounding potential adverse sexual side effects has negatively affected its public perception and may drastically reduce the number of patients who can profit from this drug.

Therefore, there is a need for alternative treatments, including physical or cosmetic treatments, supplements, and the use of herbal extracts [116,117].

The state of the art in the current treatment of HF diseases is the result of recent advances in its etiology and progression. Androgen antagonism, angiogenesis modulation, vasodilation through potassium channel opening, as well as 5-R inhibition, are the major nonsurgical pharmacological strategies employed until today [17,18]. On the other hand, the broad availability and lower costs of unconventional remedies based on the use of natural compounds has encouraged deeper investigation of their features [118,119].

Moreover, recent studies evidenced that autologous regenerative therapies such as the treatments with platelet-rich plasma (PRP), autologous growth factors, or Adipose-derived human follicle stem cells (HFSCs) are successfully used to promote hair regrowth [120,121]. Specifically, it was evidenced that PRP injections on male subjects significantly prevented HL without major side effects. Moreover, the use of autologous cell biological technique (A-CBT) based on micrografts infusions on AGA patients promoted HG [120,121]. In this context, a relevant role is played by Wnt-α-catenin-signaling pathway; specifically, the increment of Wnt signaling in FDPCs is one of the principal factors that enhanced HG [120,121]. Recent works reported the role of some natural compounds on the activation of Wnt-α-catenin signaling in the promotion of HG [122].

In the present review, we summarized the activities of seven common botanicals introduced with diet (*Panax ginseng* C.A. Mey.*, Malus pumila* Mill cultivar Annurca*, Coffee arabica* L.*, Allium sativum* L*.,* Camellia sinensis (L.) Kuntze*, Rosmarinum officinalis* L., *Capsicum annum* L.), particularly promising for their ability to reduce the rate of HL or stimulate HG. Their effects reported in a huge literature are discussed, with the limitations including the known gap occurring among different experimental strategies—in vitro, ex vivo, in vivo, and clinical trials—which makes integrative mechanistic explanations quite difficult.

## Figures and Tables

**Table 1:** Schematic representation of in vitro cellular models used to test the role of *Panax ginseng* C.A. Mey.

*Panax ginseng* C.A. Mey	In Vitro Cellular Models	Authors and Year	Results
Deciduous perennial plant belonging to the Araliaceae family. Clinically used as a herbal medicine for several millennia in East Asia.	Human dermal follicle papilla cells and keratinocytes	First Author: Shin Year: 2014	Proliferation
	Outer root sheath keratinocytes		Antiapoptotic effects

**Table 2:** Schematic representation of in vitro cellular models used to test the effects of *Malus pumila* Mill cultivar Annurca.

*Malus Pumila* Mill Cultivar Annurca	In Vitro Cellular Models	Authors and Year	Results
Cultivated in Southern Italy with a Protected Geographical Indication of the Campania region [65].	nontumorigenic human keratinocytes	First Author: Tenore Year: 2018	Increased cell survival and keratin expression.

**Table 3:** Schematic representation of in vitro cellular models used to test the role of *Allium sativum* L.

*Allium sativum* L.	In Vitro Cellular Models	Authors and Year	Results
Cultivated worldwide for use as a spice and for its medicinal properties.	Human keratinocytes.	First Author: Kim Year: 2016	Protection against UVB damage.

**Table 4:** Schematic representation of in vitro cellular models used to test the role of *Coffea arabica* L.

*Coffea arabica* L.	Cellular Models	Authors and Year	Results
Caffeine is widely consumed in foods and beverages and is also used for a variety of medical purposes. Caffeine is a xanthine (purine) alkaloid found in guarana, yerba maté, cacao, and several species used to make tea.	Hair matrix keratinocytes	First Author: Bussoletti Year: 2018	Proliferation

**Table 5:** Schematic representation of in vitro cellular models used to test the role of Camellia sinensis (L.) Kuntze.

*Camellia sinensis* (L.) kuntze	Cellular Models	Authors and Year	Results
Is a nonfermented product, obtained by harvesting fresh leaves, and is the most common in the Far East, where its consumption has very strong traditional and historical roots.	Keratinocytes	First Author: Hsu Year: 2007	Induction of p57 and caspase 14.
	Follicle dermal papilla cells	First Author: Kwon Year: 2007	Antiapoptotic effects

**Table 6:** Schematic representation of in vitro cellular models used to test the role of Rosmarinus officinalis L.

*Rosmarinus officinalis* L.	Cellular Models	Authors and Year	Results
An evergreen perennial shrub native to Europe and cultivated in many parts of the world, rosemary leaves are used as spices and flavoring agents because of the desirable flavor and antioxidant activity.	Prostate cancer cell line LNCaP.	First Author: Murata Year: 2013	Proliferation

**Table 7:** Schematic representation of in vitro cellular models used to test the role of *Capsicum annum* L.

*Capsicum annum* L.	Cellular Models	Authors and Year	Results
Used in Tunisian culinary preparations, which make them one of the most important cultivated vegetables.	Androgen-dependent prostate cancer cells (LNCaP). Human monocytic leukemia cell line THP-1.	First Author: Parisi Year: 2018	Cell viability and 5-alpha reductase activity
